# Effectiveness of cladribine therapy in patients with pulmonary Langerhans cell histiocytosis

**DOI:** 10.1186/s13023-014-0191-8

**Published:** 2014-11-30

**Authors:** Vincent Grobost, Chahera Khouatra, Romain Lazor, Jean-François Cordier, Vincent Cottin

**Affiliations:** National Reference Centre for Rare Pulmonary Diseases, Department of Respiratory Medicine, Louis Pradel Hospital; Claude Bernard Lyon 1 University, Lyon, UMR 754 France; Department of Medicine, Centre Hospitalier Universitaire Vaudois, Lausanne, Switzerland; Hospices Civils de Lyon, Hôpital Louis Pradel, Centre national de référence des maladies pulmonaires rares, Centre de compétences de l’hypertension pulmonaire, Service de pneumologie, Université de Lyon, Université Claude Bernard Lyon 1, INRA, Lyon, UMR754 France

**Keywords:** Histiocytosis, Langerhans cell granulomatosis, Cladribine, Chlorodeoxyadenosine, Pulmonary hypertension

## Abstract

**Background:**

Pulmonary Langerhans cell histiocytosis (PLCH) is a rare disorder characterised by granulomatous proliferation of CD1a-positive histiocytes forming granulomas within lung parenchyma, in strong association with tobacco smoking, and which may result in chronic respiratory failure. Smoking cessation is considered to be critical in management, but has variable effects on outcome. No drug therapy has been validated. Cladribine (chlorodeoxyadenosine, 2-CDA) down-regulates histiocyte proliferation and has been successful in curbing multi-system Langerhans cell histiocytosis and isolated PLCH.

**Methods and patients:**

We retrospectively studied 5 patients (aged 37–55 years, 3 females) with PLCH who received 3 to 4 courses of cladribine therapy as a single agent (0.1 mg/kg per day for 5 consecutive days at monthly intervals). One patient was treated twice because of relapse at 1 year. Progressive pulmonary disease with obstructive ventilatory pattern despite smoking cessation and/or corticosteroid therapy were indications for treatment. Patients were administered oral trimethoprim/sulfamethoxazole and valaciclovir to prevent opportunistic infections. They gave written consent to receive off-label cladribine in the absence of validated treatment.

**Results:**

Functional class dyspnea improved with cladribine therapy in 4 out of 5 cases, and forced expiratory volume in 1 second (FEV1) increased in all cases by a mean of 387 ml (100–920 ml), contrasting with a steady decline prior to treatment. Chest high-resolution computed tomography (HRCT) features improved with cladribine therapy in 4 patients. Hemodynamic improvement was observed in 1 patient with pre-capillary pulmonary hypertension. The results suggested a greater treatment effect in subjects with nodular lung lesions and/or thick-walled cysts on chest HRCT, with diffuse hypermetabolism of lung lesions on positron emission tomography (PET)-scan, and with progressive disease despite smoking cessation. Infectious pneumonia developed in 1 patient, with later grade 4 neutrocytopenia but without infection.

**Discussion:**

Data interpretation was limited by the retrospective, uncontrolled study design and small sample size.

**Conclusion:**

Cladribine as a single agent may be effective therapy in patients with progressive PLCH.

## Background

Langerhans cell histiocytosis (LCH) is a rare disease of unknown origin characterised by the proliferation of CD1a- and S-100-positive histiocytes, forming granulomas involving the reticuloendothelial system, lymph nodes, axial bones, skin, lungs, central nervous system, and particularly the pituitary gland with diabetes insipidus. In contrast to multi-system LCH, LCH limited to the lungs or pulmonary LCH (PLCH) is strongly associated with tobacco smoking, with more than 90% of patients who are current or former smokers versus less than 50% in multi-system LCH [1,2]. Langerhans cell proliferation in the lungs culminates in bronchiolocentric granulomas, which may cavitate into inflammatory, thick-walled cysts. Lung lesions predominate in the upper lobes with relative sparing of the bases. With disease progression, PLCH may evolve into cicatricial, fibrotic, thin-walled cysts, with irreversible lung destruction, chronic respiratory insufficiency and frequent, severe pulmonary hypertension (PH). Mutually-exclusive mutations of *BRAF* (V600E) [3], *MAP2K1* [4], and *ARAF* [5] have been identified in more than half of cases, likely contributing to clonal proliferation of Langerhans cells or precursor cells [6]. However, the potential role of corresponding inhibitors in PLCH is not known.

Smoking cessation, considered critical in PLCH management, may be followed by disease remission [7,8], although the relationship between smoking cessation and disease improvement remains unclear. PLCH recurrence, despite smoking cessation, has been reported [9]. Corticosteroids have often been given as first-line therapy in progressive PLCH, especially with prominent nodules on imaging, but without controlled proof of efficacy [10].

Cladribine or chlorodeoxyadenosine (2-CDA), a purine nucleoside analog that is directly toxic to monocytes [11], has been used successfully in multi-system LCH [12-15]. It has been reported to induce remission or improve lung disease in several adults with PLCH [16-19], and is currently being evaluated in prospective clinical trials (www.clinicaltrials.gov, NCT01473797). However, placebo-controlled trials are difficult to conduct in patients with progressive disease and severe dyspnoea. Here, we report on our single-centre experience with 5 adult patients who received cladribine therapy for progressive, symptomatic PLCH with lung function impairment.

## Methods

### Study population

This retrospective study was conducted in a tertiary centre with expertise in rare lung diseases. The diagnosis of PLCH was based on the following criteria: appropriate clinical presentation with a typical, high-resolution computed tomography (HRCT) pattern of nodules and/or cysts, relatively sparing the lung bases; or compatible clinical and imaging, and PLCH on video-assisted thoracoscopic lung biopsy. Genetic analysis of *BRAF* V600E mutation was performed in cases with available biopsy samples. The first course of treatment in patient 2 has been reported previously [16].

### Cladribine therapy

All patients received oral and written information regarding off-label cladribine given as “rescue therapy”, and their written consent was obtained in all cases prior to treatment initiation. Although treatment was not conducted in the setting of a clinical trial, and because it was prescribed off-label, particular care was taken to follow a pre-defined treatment dose and design, and to capture all potential adverse events and drug reactions. Patients were advised not to smoke at visits. Cladribine was prescribed as a single agent in all cases, especially without concomitant corticosteroid therapy or vinblastine. Treatment consisted of 3 (or 4) courses, each with 0.1 mg/kg per day of subcutaneous cladribine for 5 consecutive days at monthly intervals. It was administered intravenously in 1 case (patient 2). Oral trimethoprim/sulfamethoxazole (cotrimoxazole, 400/80 mg per day) and valaciclovir (500 mg twice a day) were systematically prescribed until 6 months after the end of cladribine therapy to prevent opportunistic infections.

### Investigations

In all patients, the following data were obtained to evaluate individual responses to therapy and benefit:tolerance as part of off-label treatment, before and 1 to 3 months after the last course of cladribine: smoking habits, World Health Organization (WHO) dyspnoea functional class, fever, weight loss, adverse events, prior and concomitant drug therapy, serum C-reactive protein (CRP) level, arterial blood gases, pulmonary function tests, including spirometry, plethysmography and low-dose HRCT. Transthoracic echocardiography was performed systematically before treatment initiation to screen for PH. Right heart catheterisation was undertaken in cases of suspected pre-capillary PH on echocardiography, e.g., with estimated systolic pulmonary artery pressure of 35 mmHg or greater. Selected cases were submitted to exploratory positron emission tomography (PET).

Institutional Review Board approval is not required for retrospective observational studies in France.

## Results

### Findings common to all 5 patients

Five patients were treated, including 1 who received therapy twice because of PLCH relapse occurring 12 months after the last cladribine dose. The diagnosis was obtained by lung biopsy in 3 cases, and by clinical and imaging criteria in the 2 other patients in whom lung biopsy was declined due to PH (patient 1) or severe lung function impairment (patient 5). *BRAF V600E* mutation was present in patients 2 and 4, absent in patient 3, and no biopsy was performed in patients 1 and 5. On imaging, a typical pattern of nodules and thick-walled cysts was seen in patients 1, 2 and 3. Patient 4 presented multiple nodules without cystic changes. A pattern with predominant thin-walled cysts was apparent in patient 5.

**Figure 1:**
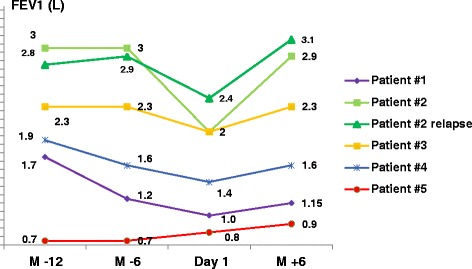
**Evolution of forced expiratory volume in 1 second (FEV1) before and after cladribine therapy in 5 patients.** M-12: 12 months before cladribine; M-6: 6 months before cladribine; Day 1 (cladribine): initiation of cladribine therapy; M + 6: 6 months after cladribine treatment.

Patient 2 was a never-smoker, whereas all the 4 others had quit smoking. Patients 1 and 4 stopped smoking 3 months before cladribine initiation, but their lung function continued to deteriorate despite smoking cessation (Figure 1). Patient 5 had quit smoking 3 years prior to treatment. Patient 3 stopped smoking between the second and third courses of cladribine. Two patients (1 and 2) had received prednisolone without success before cladribine therapy, whereas the others were treatment-naive.

**Figure 2:**
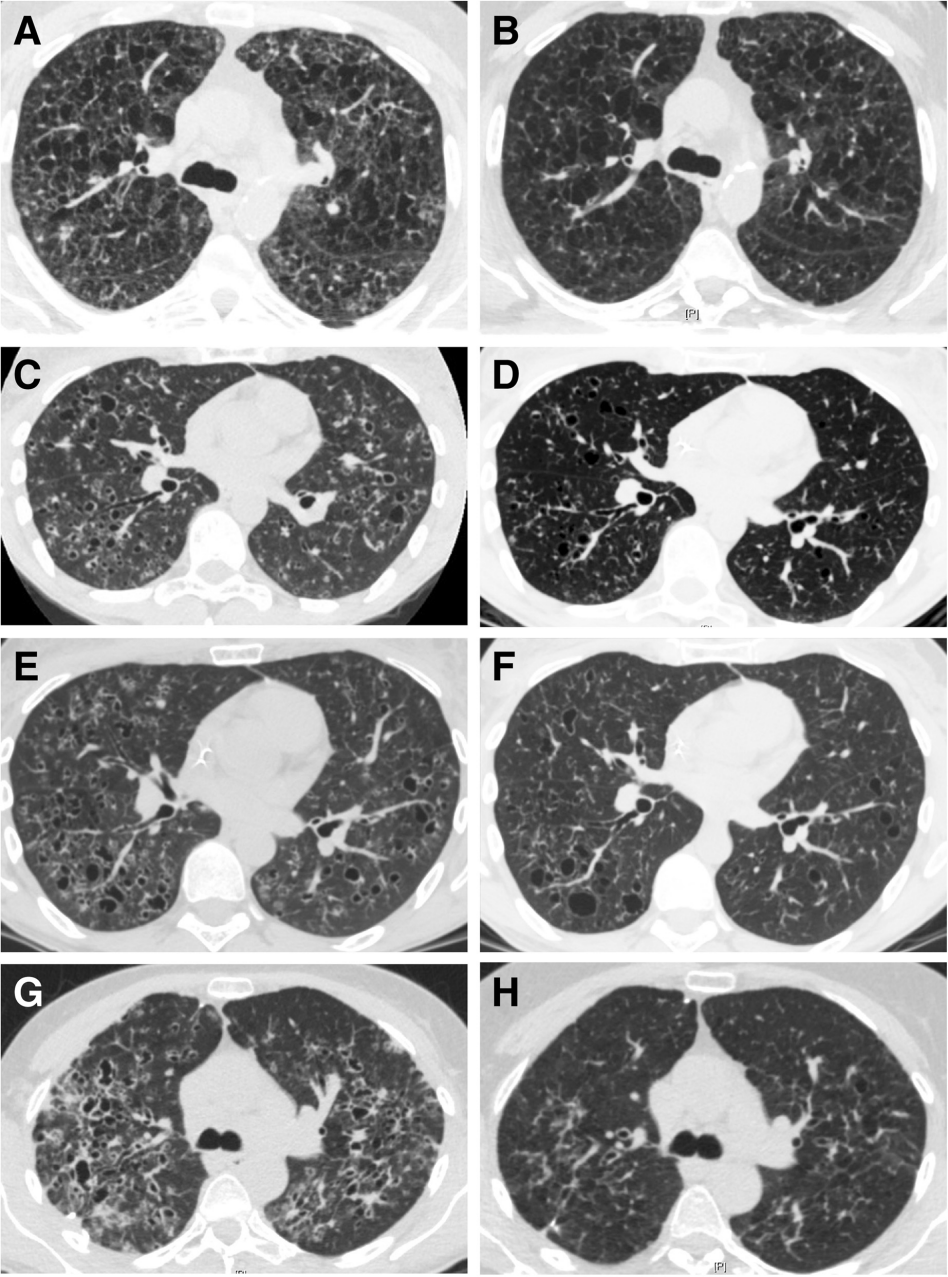
**Representative HRCT features before (A, C, E, G, I, K) and after cladribine therapy (B, D, F, H, J, L) in 3 patients. A**, **B**: patient 1; **C**, **D**: patient 2; **E**, **F**: patient 2 (relapse); **G**, **H**: patient 3.

Dyspnoea, evaluated by functional class, improved after treatment in 4 cases, and forced expiratory volume in 1 second (FEV1) increased in all cases (Figure 1). Forced vital capacity (FVC) recuperated with therapy by a mean of 414 ml (0–1,000 ml), and FEV1 by a mean of 387 ml (100–920 ml). Further pulmonary function tests and arterial blood gases are reported in Table 1. Chest HRCT features improved with cladribine therapy in 4 cases, with regression in the number and size of nodules, and decreased cyst wall thickness in all cases. Representative examples of HRCT changes are shown in Figure 2. Patients were followed for 6–48 months (median: 22 months) after cladribine therapy ended.

**Table 1:** **Patient characteristics before and after cladribine treatment**

	**WHO functional class**		**6-MWD (m)**		**FVC (l)**		**ΔFVC (ml)**	**FEV1 (l)**		**ΔFEV1 (ml)**	**FEV1/FVC (%)**		**DLco (%)**		**PaO2 (mmHg)**	
**Cladribine**	**Pre**	**Post**	**Pre**	**Post**	**Pre**	**Post**		**Pre**	**Post**		**Pre**	**Post**	**Pre**	**Post**	**Pre**	**Post**
***Patient 1 *** ***M, 45 y***	IV	III	60	220	2.67 (56%)	2.93 (62%)	+260	1.00 (28%)	1.15 (32%)	+150	38	37	15	16	60 (3 l)	65 (2 l)
***Patient 2 *** ***F, 37 y***	II	0	NA	NA	2.54 (66%)	3.54 (95%)	+1,000	1.98 (61%)	2.90 (90%)	+920	95	82	58	NA	NA	NA
**Patient 2 ** ***F, 37 y relapse***	II	0	NA	NA	3.33 (87%)	4.0 (110%)	+670	2.43 (75%)	3.10 (95%)	+670	72	79	52	70	NA	NA
**Patient 3 ** ***F, 55 y***	II-III	I	NA	NA	2.88 (112%)	3.30 (126%)	+420	1.96 (89%)	2.30 (104%)	+340	69	71	67	62	82.5	NA
**Patient 4 ** ***M, 50 y***	II	II	495	NA	4.30 (98%)	4.43 (101%)	+130	1.40 (41%)	1.61 (47%)	+210	32	36	66	68	79.5	91.5
**Patient 5 ** ***F, 40 y***	II-III	II-III	267	282	2.6 (78%)	2.6 (79%)	+0	0.8 (29%)	0.9 (31%)	+100	29	18	30	28	82.5	84

NA: not available; 6-MWD: 6-minute walk distance; DLco: diffusing capacity of carbon monoxide, FEV1: forced expiratory volume in 1 second; FVC: forced vital capacity; Δ: variation of FVC and FEV1 before and after cladribine treatment; PaO2: pulmonary arterial tension in blood gases, WHO: World Health Organization.

Results were obtained within hours or days before the first course of cladribine therapy (“Pre”), then 1 month (up to 3 months) after the last cladribine dose (“Post”). Volumes are indicated after inhalation of short-acting bronchodilators.

### Individual observations

Patient 1: A 55-year-old man, current smoker, with a history of 50 pack-years, presented in November 2010 with dyspnoea functional class III. Pulmonary function tests indicated an obstructive ventilatory pattern with 16% diffusion capacity of carbon monoxide (DLco). At echocardiography, estimated systolic pulmonary arterial pressure was 86 mmHg. Right heart catheterisation disclosed pre-capillary PH, with mean pulmonary arterial pressure (mPAP) of 45 mmHg, pulmonary artery wedge pressure (PAWP) of 13 mmHg, cardiac index of 3 l/min/m^2^, and pulmonary vascular resistance (PVR) of 428 dyn.s^−1^.cm^−5^. Serum levels of NT-pro-brain natriuretic peptide and CRP were 417 pg/ml and 4 mg/l, respectively. No significant hypermetabolism was seen on PET-scan. PaO2 was 66 mmHg under 3 l/min nasal oxygen, and long-term nasal supplemental oxygen was initiated. The patient was advised to quit smoking, and bosentan was started (62.5 mg, then 125 mg twice a day). Four months later, despite decreased smoking, from 20 to 5 cigarettes per day, dyspnoea (functional class IV) and lung function worsened. Right heart catheterisation demonstrated hemodynamic improvement (mPAP: 45 mmHg, PAWP: 11 mmHg, PVR: 337 dyn.s^−1^.cm^−5^, cardiac index: 4 l/min/m^2^). The patient definitively stopped smoking. In April 2011, prednisolone (1 mg/kg per day) was initiated, with gradual tapering to 0.5 mg/kg per day over 3 months. In July 2011, lung function continued to deteriorate, with further decrease in FEV1 of 200 ml. Severe functional class IV dyspnoea persisted. Chest HRCT revealed a typically-diffuse nodular pattern with thin-walled cysts. Prednisolone was discontinued, and cladribine was initiated. After 4 courses of subcutaneous cladribine therapy with no significant adverse reactions, dyspnoea, lung function, PaO2 (Table 1), and HRCT findings (Figure 2) improved. Hemodynamic values rallied further (mPAP: 40 mmHg, PAWP: 11 mmHg, PVR: 240 dyn.s^−1^.cm^−5^, cardiac index: 4.87 l/min/m^2^). Unfortunately, the patient resumed smoking 6 months after cladribine therapy. PLCH relapsed, with increased dyspnoea and deterioration of lung function.

Patient 2: A 37-year-old woman, non-smoker, was seen in 2006 for dry cough, dyspnoea functional class II, fever, night sweats, and weight loss. She had a medical history of operated benign prolactinoma, with no sign of pituitary LCH at histology. HRCT was typical for diffuse nodular PLCH, which was confirmed by surgical lung biopsy. Pulmonary function tests showed a restrictive pattern with reduced DLco. CRP level was 45 mg/l. PET-scan disclosed diffuse hypermetabolism of lung parenchyma. The patient received prednisolone (0.5 mg/kg per day from October to March 2007), with no change in clinical, functional, or imaging manifestations. She then received 4 courses of intravenous cladribine (from March to July 2008), which was well tolerated. All symptoms had resolved 4 months after treatment ended. CRP level was 1 mg/l. Lung function and HRCT findings improved. No hypermetabolism was found on PET-scan. In July 2009, PLCH relapsed with dry cough, dyspnoea functional class II, CRP level of 25 mg/l, and lung function deterioration (Table 1). Furthermore, a subcutaneous mass of the vertex had developed which, on MRI, corresponded to a cranial bone tumour without cerebral involvement. PET-scan showed diffuse lung hypermetabolism, with focal hypermetabolism of the cranial bone mass (SUV max was 3.9). The tumour was removed surgically. Histology disclosed a LCH bone lesion not invading the meninges, with positive CD1a and S-100 staining. The patient received 3 additional courses of intravenous cladribine with good tolerance. She was asymptomatic at the end of treatment. PET-scan showed no hypermetabolism. HRCT findings improved significantly. CRP level was 4 mg/l. Disease remission persisted to the last visit 4 years after the end of treatment.

Patient 3: A 55-year-old woman, smoker (40 pack-years), presented with dyspnoea functional class II-III and chronic cough. CRP level was elevated at 83 mg/l. Chest HRCT showed a typical PLCH pattern, with numerous small nodules and thick-walled cysts. Pulmonary function tests indicated isolated reduction of DLco (72% of predicted), with normal spirometry – FVC: 2.47 l (95%); FEV1: 1.96 l (89%); FEV1:FVC: 75%; total lung capacity (TLC): 4.6 l (102%). Echocardiography was normal. PET-scan demonstrated diffuse lung hypermetabolism without extra-pulmonary involvement (SUV max: 5.5). Despite smoking cessation, FEV1 declined by 280 ml over 6 months of follow-up. Right lung surgical biopsy confirmed the PLCH. The patient then received 2 courses of subcutaneous cladribine, in March and April 2013. In May 2013, however, she presented right pneumothorax needing chest tube drainage, with recurrence in June 2013 requiring thoracic surgery with pleurodesis. A third course of cladribine therapy was administered in July 2013. In October 2013, she had dyspnoea functional class I, without cough. CRP level was 3 mg/l. HRCT findings showed significantly decreased nodules, with complete resolution of several thick-walled cysts (Figure 2). PET-scan after treatment found no significant hypermetabolism. Pulmonary function tests disclosed improvement of FEV1 (+300 ml) and FVC (+420 ml), with unchanged DLco.

Patient 4: A 50-year-old current smoker (40 pack-years) was admitted in June 2011 for spontaneous left pneumothorax, night sweats, and weight loss (−7 kg). His past medical history included chronic obstructive pulmonary disease (GOLD class III) with emphysema, treated with inhaled tiotropium, budesonide and formeterol. Chest HRCT revealed diffuse micronodules without cysts, and “atypical emphysema”. PET-scan showed no pulmonary hypermetabolism. Lung surgical biopsy demonstrated PLCH, with involvement of the bronchioles, and emphysematous changes. Echocardiography was normal. Although pneumothorax had resolved, dyspnoea functional class II persisted, and pulmonary function tests indicated an obstructive pattern. Despite complete smoking cessation in March 2013, lung function continued to deteriorate, with FEV1 decreasing by 200 ml and FVC by 100 ml over 6 months, and dyspnoea functional class II. CRP level was 22 mg/l. Chest HRCT was unchanged. Cladribine therapy was initiated. Seven days after the first course, the patient presented infectious pneumonia, without neutropenia, and was successfully treated with intravenous ceftriaxone, but required transfer to the intensive care unit for 48 hours. The second course was complicated by severe neutropenia (grade 4) on day 12, without fever or infection. A 25% dose reduction of cladribine was decided for the third course, which was well tolerated. Cladribine therapy was followed by general improvement, with unchanged dyspnoea functional class II. FVC and FEV1 values increased by 130 ml and 200 ml, respectively. CRP level was 7 mg/l. HRCT findings improved significantly. PET-scan showed no hypermetabolism.

Patient 5: A 40-year-old woman, ex-smoker (20 pack-years up to 3 years earlier), presented dyspnoea functional class II-III in association with diabetes insipidus. MRI showed isolated pituitary stalk enlargement. Hormonal analyses indicated isolated central diabetes insipidus, which was treated with desmopressin. Chest HRCT revealed thin-walled cysts and emphysematous-like lesions, without nodules or thick-walled cysts. PET-scan showed no significant hypermetabolism. Echocardiography was normal. Lung functional tests disclosed a severely-obstructive pattern: FVC: 2.6 l (80% of predicted), FEV1: 0.8 l (29%), FEV1:FVC: 29%, TLC: 6.7 l (134%), DLco: 30%. Αlpha-1 antitrypsin level, CRP and protein electrophoresis were normal. A diagnosis of PLCH was made. Treatment was attributed to long-term lung function decline despite smoking cessation and high-dose inhaled bronchodilators, with a mean decrease of 300 ml/year and 233 ml/year in FEV1 and FVC, respectively, over a 3-year period. The patient received 4 courses of subcutaneous cladribine therapy, with good tolerance. No improvement was observed in dyspnoea, arterial blood gases, and HRCT findings, but FEV1 recovered by 100 ml (Table 1, Figure 1), and the FVC decline was stopped. The patient was considered stable at the last visit, 2 years after the last cladribine dose, and evaluation for lung transplantation was postponed.

## Discussion

We report on 5 patients successfully treated with cladribine for progressive PLCH with obstructive ventilator pattern. Cladribine treatment arrested the decline in FEV1 and was followed by either stabilization (patient 5) or improvement (patients 1 to 4) of airflow limitation. Although additional evidence is needed through placebo-controlled randomised trials, these observations suggest that cladribine, as a single agent, may be considered as rescue therapy in subjects with severe disease worsening despite smoking cessation and, in some cases, despite oral corticosteroids. All patients were younger than 55 years, and at least 2 of them (patients 1 and 5), in whom lung transplantation would have been considered over the mid-term, were stabilised after cladribine therapy, and transplantation was not deemed necessary.

The outcome of treatment may be more evident in individuals with moderately severe obstructive lung disease due to PLCH and not confounded by tobacco smoking, but disease stabilisation and some clinical benefits were also observed in patients 4 and possibly 5 with severe chronic obstructive ventilatory pattern related to both PLCH and history of tobacco-smoking. *BRAF V600E* mutation in 2 patients (2 and 4) did not seem to affect the response to cladribine. Three of 5 patients (3, 4, and 5) were treatment-naive, whereas cladribine was given after unsuccessful first-line prednisolone therapy in the first 2 cases who had rapidly progressive PLCH despite smoking cessation. Encouraging results in the first treated patients led us to consider potential cladribine therapy earlier in disease management of more recent cases. Given the currently low level of evidence, we think that treatment decisions should probably be based on the degree of lung impairment and on the rapidity of disease worsening. Interestingly, cladribine was successful in 1 patient with relapsing pulmonary disease (patient 2).

Chest HRCT showed improvement in most cases, with significantly decreased nodules and thick-walled cysts. No significant change was observed in HRCT findings in patient 5 with thin-walled cysts and emphysematous alterations consistent with relatively-stable lung function in this patient, likely corresponding to non-inflammatory disease. The observation is consistent with a pathological-radiological correlation study, suggesting that nodules on imaging generally correspond to an active granulomatous process at pathology, whereas cavitary lesions on imaging usually reflect still active inflammatory cavitary granulomas or cicatricial fibrous cysts [20]. Cladribine therapy may, therefore, be particularly effective in PLCH subjects with a predominant pattern of diffuse nodules and/or thick-walled cysts on HRCT.

Hypermetabolism on PET-scan has been reported in PLCH patients, with more frequent and higher metabolism early in the course of the disease and in those with nodular lung lesions as well as thick-walled cysts on chest imaging [21]. In the present series, patients 2 and 3, who had positive PET findings and predominantly diffuse nodules as well as thick-walled cysts at HRCT, were also those with the greatest improvement in lung function after cladribine therapy. Whether PET may be undertaken to identify patients with active nodular lung lesions and predict better responses to cladribine therapy remains to be investigated prospectively.

Cladribine further improved haemodynamics in 1 patient with severe PH associated with PLCH, consistent with reports showing involvement of pulmonary arteries and veins in granulomas and remodeled pulmonary arteries within Langerhans cell granulomas [22,23], as also seen in sarcoidosis.

Cladribine is a chemotherapeutic agent with potential toxicity, including infections related to immunosuppression, cytopenia, vomiting, diarrhoea, increased liver enzymes, rash, purpura and, less frequently, neurotoxicity. Treatment of our patients was remarkably uneventful, with the notable exception of 1 patient (#4) who had infectious pneumonia with bronchospasm successfully controlled by antibiotic therapy. Later, the patient had grade 4 neutrocytopenia without infection, which did not relapse after dose adjustment. Patients systematically received preventive therapy with oral trimethoprim/sulfamethoxazole and valaciclovir, and were monitored for cytopenia. The subcutaneous route was preferred to intravenous injection in most cases for convenience and good tolerance.

Limitations of this study include small sample size related to low PLCH prevalence with, furthermore, a small proportion of patients incurring rapidly progressive disease refractory to smoking cessation and/or corticosteroids. Although placebo-controlled randomised trials would ideally be desirable for this condition, patient inclusion has recently proven to be challenging (www.clinicaltrials.gov, NCT01473797), as those at risk of progression to chronic respiratory insufficiency and their doctors are understandably reluctant to participate. Despite the retrospective design, patients in our study were monitored very closely, ensuring exhaustive capture of adverse events and outcome variables. Tolerance and safety were generally acceptable, with a favourable benefit:risk ratio, but long-term follow-up is warranted regarding potential secondary malignancies that could be facilitated by cladribine. Tobacco-smoking was a potentially-confusing factor in patient 3, who quit the habit between the second and third courses of cladribine. Patients received 3 to 4 courses of cladribine, but the optimal number of courses, and the potential utility of maintenance therapy, need to be explored further.

## Conclusion

In conclusion, cladribine, as a single agent, may be effective therapy in PLCH patients. It may be considered, especially in subjects with progressive disease despite smoking cessation, with nodular lung lesions and/or thick-walled cysts on chest HRCT, and with diffuse hypermetabolism of lung lesions on PET-scan.
